# Modulation of visual processing of food by transcutaneous vagus nerve stimulation (tVNS)

**DOI:** 10.1007/s11682-020-00382-8

**Published:** 2020-09-14

**Authors:** Helena Alicart, Marcus Heldmann, Martin Göttlich, Martina A. Obst, Marc Tittgemeyer, Thomas F. Münte

**Affiliations:** 1 Cognition and Brain Plasticity Group, Bellvitge Biomedical Research Institute (IDIBELL), 08097, L’Hospitalet de Llobregat, Barcelona, Spain; 2 Department of Cognition, Development and Educational Psychology, Institute of Neurosciences, Campus Bellvitge, University of Barcelona, 08097, L’Hospitalet de Llobregat, Barcelona, Spain; 3 Department of Neurology, University of Lübeck, 23538 Lübeck, Germany; 4 Center of Brain, Behavior and Metabolism (CBBM) , University of Lübeck , Ratzeburger Allee 160, Lübeck, 23562 Germany; 5 Translational Neurocircuitry Group, Max-Planck Institute for Metabolism Research, Cologne, 50931 Cologne, Germany; 6 Cluster of Excellence in Cellular Stress and Aging-Associated Disease (CECAD) , Cologne, 50931 Germany

**Keywords:** Vagus nerve stimulation, Ingestive behavior, Food, Functional MRI

## Abstract

Present project is concerned with the possibility to modulate the neural regulation of food intake by non-invasive stimulation of the vagus nerve. This nerve carries viscero-afferent information from the gut and other internal organs and therefore serves an important role in ingestive behavior. The electrical stimulation of the vagus nerve (VNS) is a qualified procedure in the treatment of drug-resistant epilepsy and depression. Since weight loss is a known common side effect of VNS treatment in patients with implanted devices, VNS is evaluated as a treatment of obesity. To investigate potential VNS-related changes in the cognitive processing of food-related items, 21 healthy participants were recorded in a 3-Tesla scanner in two counterbalanced sessions. Participants were presented with 72 food pictures and asked to rate how much they liked that food. Before entering the scanner subjects received a 1-h sham or verum stimulation, which was implemented transcutanously with a *Cerbomed NEMOS*® device. We found significant activations in core areas of the vagal afferent pathway, including left brainstem, thalamus, temporal pole, amygdala, insula, hippocampus, and supplementary motor area for the interaction between ratings (high vs low) and session (verum vs sham stimulation). Significant activations were also found for the main effect of verum compared to sham stimulation in the left inferior and superior parietal cortex. These results demonstrate an effect of tVNS on food image processing even with a preceding short stimulation period. This is a necessary prerequisite for a therapeutic application of tVNS which has to be evaluated in longer-term studies.

The vagus nerve is composed of about 80% afferent fibers and provides bidirectional information between the brain and peripheral organs (de Lartigue 2016). It carries visceral, somatic and taste information, which makes the vagus nerve relevant to food intake behavior. Vagal activity is mediated by stretch and mechanoreceptors as well as by stomach and gut hormone signaling following food consumption including leptin, ghrelin, cholecystokinin (CCK), and glucagon like peptide 1 (GLP-1) among others (Williams et al. 2016; for reviews, see: Woods 1998; Bray 2000; Morton et al. 2006, 2014; Berthoud 2008; Page and Kentish 2017; de Lartigue 2016). Satiety signals are conveyed to the central nervous system (CNS) through afferent fibers of the vagus nerve, which terminate mainly in the nucleus of the solitary tract (NTS), an important relay center for a variety of vital functions located in the dorsal vagal complex of the medulla oblongata (Sawchenko 1983; Jean 1991). The NTS is the only visceral afferent relay station in the human brainstem and displays the primary input area for gustatory sensing (Bradley 2007; Veldhuizen et al. 2011). The rostral portion of the NTS receives gustatory information via the cranial nerves VII and IX, as well as fibers of the auricular branch of the vagus nerve (de Lartigue 2016; de Lartigue and Diepenbroek 2016), whereas the more medially and caudally located parts of the NTS receive mainly viscerosensory information via baro- and chemoreceptors. The main ascending gustatory pathway is defined by collaterals of rostral NTS neurons projecting to the ipsilateral parabrachial nuclei (PbN; Morton et al. 2014), and to several regions in the brainstem, limbic system and forebrain (Ruffoli et al. 2011; and Berthoud 2008 for reviews). Among brainstem regions, NTS projections reach the locus coeruleus (LC) and raphe nuclei (Saper and Loewy 1980; Van Bockstaele et al. 1999; Groves and Brown 2005; and Krahl and Clark 2012 for reviews), which provide widespread noradrenergic and serotonergic innervation of the brain. These nuclei are believed to play a key role in the mechanisms of action underlying vagus nerve stimulation (VNS; Henry 2002; Groves et al. 2005; Fornai et al. 2011; Yakunina et al. 2017). The PbN itself sends efferents to thalamic nuclei, the hypothalamus, the primary gustatory cortex (anterior insula and the adjacent opercular cortex; Veldhuizen et al. 2011) and also to limbic regions like the central nucleus of the amygdala and the bed nucleus of the stria terminalis. Several of these structures have been reported to project back to the PbN, indicating a close relationship of the gustatory pathway, hypothalamic energy homeostasis and limbic circuit.

The neural control of feeding has previously focused mainly on signaling mechanisms associated with the hypothalamus, the major center in the brain that regulates body weight homeostasis. Along with the systems monitoring food intake to maintain energy balance, non-homeostatic appetitive eating is considered part of reward-related behavior (Berthoud 2006; Lutter and Nestler 2009; Castro et al. 2015; Shechter and Schwartz 2018).

Because of this central position in several important brain networks, the vagus nerve has been a target for therapeutic trials. Invasive VNS with an implanted device in the cervical region has been used since the late 1980s for the treatment of drug-resistant epilepsy and depression (reviews in George et al. 2000; and Chae et al. 2003). Moreover, VNS has been studied for the treatment of other psychiatric disorders such as dementia, schizophrenia or anxiety disorders (Groves and Brown 2005; Vonck et al. 2014; and Cimpianu et al. 2017). In addition to the role of vagus nerve in food intake behavior, evidence of the potential therapeutic mechanism of VNS for the treatment of obesity comes from studies with depressed patients (Pardo et al. 2007) and patients with pharmacoresistant epilepsy (Burneo et al. 2002) with implanted devices showing weight loss after chronic VNS (from 6 months to 2 years of treatment). In the same vein, (Bodenlos et al. 2007a; 2007b) found that depressed patients receiving VNS showed a reduction in craving and arousal ratings for sweet foods. Similarly, results from VNS in animal studies have revealed a decrease in food consumption (Val-Laillet et al. 2010; Gil et al. 2011), weight loss or decreased weight gain (Roslin and Kurian 2001; Val-Laillet et al. 2010; Gil et al. 2011; Li et al. 2015) and a reduction in the cravings for sweet foods (Val-Laillet et al. 2010).

>More recently, a less invasive method was been developed for the stimulation of the vagus nerve, with a proven comparable effectiveness to invasive VNS (Kraus et al. 2007; Ellrich 2011; Stefan et al. 2012; Frangos et al. 2015; Safi et al. 2016; Yakunina et al. 2017). Kraus et al. (2007) were the first to investigate the effect of VNS with a transcutaneous approach (tVNS), with an electrode placed in the inner side of the tragus of the left ear that delivered electrical impulses at the auricular branch of the vagus nerve. Other studies have used tVNS in the inner tragus and in the cymba conchae of the left ear in conjunction with fMRI, with subsequent activations in LC and NTS as compared with sham stimulation (Dietrich et al. 2008; Frangos et al. 2015; Safi et al. 2016; Yakunina et al. 2017) and BOLD signal increases in brain regions such as the thalamus, postcentral gyrus, prefrontal cortex, amygdala, insula or nucleus accumbens among others (Dietrich et al. 2008; Frangos et al. 2015). Concerning the cymba conchae, anatomical postmortem studies on the auditory branch of the vagus nerve (ABVN) have confirmed the termination of the ABVN in the cymba conchae at the outer ear, thus being the most appropriate place for the stimulation (Peuker and Filler 2002; Safi et al. 2016). Besides, the existence of myelinated axons in the ABVN has been also reported, which together with the aforementioned fMRI studies, validate the suitability of this less intrusive technique as an alternative to invasive VNS.

The goal of this study was to assess the effect of tVNS on the afferent vagal pathway with reference to food stimuli processing. To address this question, we used food pictures in order to assess brain responses after verum and sham tVNS in healthy participants in a single-blind placebo-controlled design. We hypothesized that a modulatory effect of tVNS on the valuation of food pictures would manifest itself in the interaction between stimulation condition (sham vs. verum) and liking ratings (high vs. low). The demonstration of such interaction effects is therefore the most important outcome of this study. As a secondary hypothesis, we expected to find lower liking ratings to food images following verum in comparison with sham stimulation. It is known that foods are considered more pleasant when people are hungry, and a reduction in the perception of food reward value is a direct effect of satiety (Cabanac 1979; Mehta et al. 2012). Finally, we also hypothesized to see a decrease of the food consumption following tVNS as compared to sham stimulation as a result of the expected satiety-mimicking effect of the stimulation of the vagus nerve (Gil et al. 2011).

## Methods

### Participants

Twenty-one healthy right-handed women (Caucasian, upper-middle class, undergraduate students, mean ± SD age, 23.52 ± 2.1 years old) with a normal body mass index (BMI; 18.5 to 25) participated in the study. None of the participants was following a dietary restraint (please see the section *Questionnaires* (hereunder) for the eating behavior assessment). All procedures were approved by the ethical committee of the University of Lübeck, and informed consent was obtained from all participants. The study was performed in agreement with the Declaration of Helsinki. All participants received verum and sham stimulation in a counterbalanced placebo-controlled single-blind study design, with a minimum period of 1 week between sessions. We also controlled for day of the menstrual cycle in order to avoid a confounding hormonal effect. There were no differences between the actual day of the menstrual cycle between both verum and sham stimulation (*t*(16) = 0.95, *p* = 0.36; three participants were taking oral contraceptives). For both sessions, participants were instructed to be fasting from the previous afternoon (6 p.m.) and that this could be verified by blood test. Only water and/or unsweetened tea were allowed in the morning. Scanning sessions started at 9 a.m. or 10 a.m. Each participant had both sessions at the same hour of the day. Therefore, there were no differences between sessions with respect to the hours they had been fasting (*t*(19) = 1.14, *p* = 0.27). Also, all measurements were performed in the morning to avoid the effect of the circadian modulation of the hormones regulating food intake. After the scanning sessions, participants were offered a complete standardized vegetarian breakfast, with the purpose to explore a possible reduction on food consumption after receiving verum stimulation. Breakfasts had enough variety and quantity of foods to ensure that participants’ ad libitum eating behavior could be adequately assessed. Each breakfast contained 3800 cal, 130 g of proteins, 150 g of fat and 450 g of carbohydrates. The consumed food was quantified.

### Questionnaires

In the first session two questionnaires related to food intake behavior and one inventory of depressive symptomatology were given. The *Dutch Eating Behavior Questionnaire* (DEBQ; Van Strien et al. 1986) is a 33-item questionnaire used for measuring trait eating behaviors. It has three subscales measuring the constructs of emotional eating (13 items), external eating (10 items) and restrained eating (10 items). Responses are given via a 5-point Likert scale ranging from “never” (1) to “very often” (5). The *Fragebogen zum Essverhalten,* the German Version of the Three-Factor-Eating-Questionnaire (Stunkard and Messick 1985), consists of three factors: cognitive restraint of eating, disinhibition and hunger. Some questions were answered “true” or “not applicable” (1 or 0 points respectively) and questions with 4 answer options were given 1 point for “always” and “often” and 0 points for “rarely” or “never”. Finally, participants were also asked to answer the *Quick Inventory of Depressive Symptomatology-Self-Report* (QIDS-SR30; Rush et al. 1996), in order to exclude significant depressive symptomatology. This scale consists of 30 items with 4 possible answers from 0 to 3, and participants are asked to choose the one that best describes themselves over the last 7 days. Mean scores and S.D. for the three questionnaires are shown in Table 1.

**Table 1:** Descriptive data of the sample: age, body mass index (BMI) and scores of the three questionnaires (overall and subscales)

*Participants*	Mean	*SD*
Age	23.52	2.09
BMI	21.3	2.22
*Questionnaires*		
DEBQ	2.62	0.45
DEBQ Restrained	2.72	0.91
DEBQ Emotional	2.16	0.74
DEBQ External	3.08	0.44
FEV	18.02	7.30
FEV Cognitive Control	7.30	2.81
FEV Disinhibition	6.53	2.17
FEV Hunger	4.85	2.28
QIDS-SR30	7.35	5.95

### Stimulation procedure

**Fig. 1:**
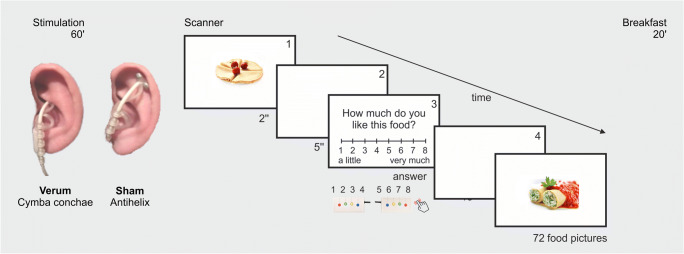
Stimulation sites for verum and sham stimulation (60 min). Illustration of the task: Picture presentation (1). 5-s delay (2). Liking ratings (3; cVAS from 1 to 8). Delay (4)

Before entering the scanner, subjects received a 1-h verum or sham stimulation in two counterbalanced sessions. tVNS was implemented with a *Cerbomed NEMOS*® device placed in the cymba conchae at the left outer ear for verum stimulation (where the auricular branch of the vagus nerve traverses), and in the scaphoid fossa of the left ear for sham stimulation (with no access to the vagal nerve). The similarity between the locations was chosen in order to keep the participants blind with regard to stimulation condition (see Fig.1). The stimulation intensity was the same for all the participants. We started with the lowest intensity, increasing it gradually until the participant had the perception of the electrical stimulation. This was done to convince the participants that they actually received stimulation in each session. Then, the intensity was adjusted to 0.6 mA for all participants and sessions. Previous studies on tVNS reported an adjustment of the stimulation intensity to the participants’ perception threshold (Kraus et al. 2007; Huang et al. 2014; Clancy et al. 2014; Yakunina et al. 2017). On the other hand, other studies on cognitive benefits of VNS have reported a U-shape curve regarding the stimulation intensity where intermediate intensities (∼0.5 mA) led to a noradrenergic facilitation of long-term potentiation in the hippocampus (for review see Vonck et al. 2014). The stimulation frequency and pulse width were set to 25 Hz and 250 μs, respectively. Stimulation was on for 30 s followed by a pause of 30 s.

### Experimental paradigm – Task

After 1 h of verum or sham stimulation, participants were placed in the scanner. While recording functional MRI participants were presented with 72 food pictures in a random order. Food pictures included palatable food, sweet and savory, high and low caloric. Images were presented for 2 s, centered on the screen. After 5 s, a computerized visual analogue scale (cVAS) from 1 to 8 appeared on the screen and participants were asked to rate how much they liked that food. Images were presented every 20 s.

### Data acquisition

Whole brain fMRI data were obtained by using a 3.0 Tesla scanner equipped with 64 channel phase array head coil (Siemens MAGNETOM Skyra) located at the Center of Brain, Behavior and Metabolism, Lübeck, Germany). For each session, functional and structural measurements were carried out. Functional task measurements comprised three runs of 240 scans (Gradient Echo EPI; repetition time = 2090 ms; echo time = 25 ms; flip angle = 80^o^; voxel size = 3x3x3; 42 slices; matrix size = 64 × 64; interleaved acquisition). 24 food images were presented in each run. High resolution T1-weighted anatomical images were acquired in the first and second session respectively (192 slices, image matrix 256 × 256, 1x1x1 mm^3^, TR 1900 ms, TE 2.44 ms, flip angle 9°).

### Data analysis

The analysis of the ratings to the food images inside the scanner using a cVAS from 1 to 8 was performed using the non-parametric Wilcoxon signed-rank test for related samples. For reaction time and fMRI data analyses, ratings were divided into “high” and “low” by performing a median split for each participant and session. Mean subject values of the reaction times measured in milliseconds were entered into a 2 × 2 ANOVA with the factors stimulation (verum or sham) and ratings (high or low). Post-hoc paired samples *t*-tests were performed for the factors showing significant differences. Possible differences between sessions in the scores of the three VAS outside the scanner before and after having breakfast were also assessed with the Wilcoxon signed-rank tests for related samples. Behavioral analyses were performed using SPSS 18.0 software (SPSS Inc., Chicago, USA 2009).

### fMRI data analysis

fMRI data were analyzed using the Statistical Parameter Mapping software (SPM12, Wellcome Department of Imaging Neuroscience, University College, London, UK. www.fil.ion.ucl.ac.uk/spm/). Preprocessing included slice time correction, realignment and normalization to MNI template space. Then, the images were smoothed with an 8 mm Gaussian kernel. One participant was excluded due to extensive head movement (>3 mm displacement from the first image or rotation >3 degrees). There were no differences in movement between the first and second sessions.

To investigate potential VNS-related changes in the cognitive processing of food-related items we performed a 2 × 2 flexible factorial analysis with the factor ratings (high and low) and session (verum and sham stimulation). Only those cluster corrected at FWEc = 0.05 (cluster defining threshold *p* < 0.005 with 20 voxels extent) are reported. Anatomical areas were identified using the Automated Anatomical Labeling Atlas (Tzourio-Mazoyer et al. 2002) included in the xjView toolbox (http://www.alivelearn.net/xjview8/).

In addition to the whole brain analysis, we performed a ROI analysis in areas that were specified a priori because they have been reported to be sensitive to either VNS/tVNS stimulation or to food rewards in previous studies (Bohning et al. 2001; Lomarev et al. 2002; Kringelbach 2005; Wang et al. 2006; Dietrich et al. 2008; Rolls and Grabenhorst 2008; Castro et al. 2015; Alves et al. 2015; for a review see Ferrario et al. 2016). The following ROIs were defined: hypothalamus, nucleus accumbens, hippocampus, orbitofrontal cortex (OFC) and dopaminergic midbrain. Individual beta coefficients (extracted from the subjects’ first level fMRI analysis) were calculated for each participant by averaging the mean signal within the significant ROIs (creating a 5 mm radius sphere around the clusters peak).

## Results

### Behavioral results

Online ratings to food pictures had a median = 6, interquartile range (IQR) = 1 for verum stimulation session and a median = 6, IQR = 1 for sham stimulation session. Wilcoxon signed-rank test for related samples revealed marginal significant differences between sessions (*Z* = 1.90, *p* = .06), with a higher number of cases with higher median values for sham stimulation session.

Our participants were asked to be fasting from the previous afternoon (6 p.m.). Mean ± SD hours of fasting for each session were 17.5 ± 0.5 for real stimulation and 17.5 ± 0.6 for sham stimulation (see Table 2). After each scanning session participants had a complete breakfast, which was weighed before and after eating. No differences in food intake, fat, proteins, carbohydrates (all measured in grams) or calories (kcal) were present between stimulation and sham sessions.

**Table 2:** Food intake and subjective ratings

	Verum stimulation		Sham stimulation		Sing.
	*Mean*	*SD*	*Mean*	*SD*	
Calories (Kcal)	982.5	326.9	973.7	33.19	n.s.
Proteins (g)	37.6	14.5	39.2	22.1	n.s.
Fat (g)	36.6	18.3	37.6	19.9	n.s.
Carbohydrates (g)	121.2	31.5	119.3	36.4	n.s.
Grams consumed	595.8	209.7	567.3	220.9	n.s.
	*Median*	*IQR*	*Median*	*IQR*	
Online ratings (VAS)	6	1	6	1	m.s.*
Hunger (VAS)	7	1.75	7	0.75	n.s.
Desire (VAS)	7	1	7	1	n.s.
Like food (VAS)	7	1.75	6	1.75	n.s.
Satiety (VAS)	7	1	7	2	n.s.

*Marginally significant

Before having breakfast, participants were asked to rate on three visual analogue scales from 1 (a little) to 8 (very much) referring to how hungry they were, how much they liked the food (breakfast) and how strong was their desire to eat. After eating, they were also asked to rate their satiety on a similar scale. Mean scores and SD are shown in Table 2. There were no differences in subjective ratings between verum and sham stimulation (*Z* > 1.40, *p* > 0.16 for all comparisons).

### fMRI results

#### Whole brain analysis

In order to investigate potential VNS-related changes in the cognitive processing of food-related items we performed a 2 × 2 flexible factorial analysis with the factors ratings (high and low) and session (verum and sham stimulation).

Main effects for the factor ratings and main effects for the factor session are shown in Table 3. With regard to the factor session, fMRI enhancements for the picture presentation for verum over sham stimulation were found in left inferior and superior parietal lobule (FWE corrected).

**Table 3:** Main effects of verum vs sham stimulation and high vs low ratings

Anatomical area	Cluster level			
	Coordinates	*FWEc*	Cluster size	*F*
*Verum* vs *sham stimulation*				
Parietal_Inf_L	−27 –45 54	0.047	50	15.78
Parietal_Sup_L	−36 –48 63			11.10
*High* vs *low ratings*				
Precentral_L	−33 –21 54	<.0001	4851	179.48
Postcentral_L	−45 –27 63			105.29
Parietal_Sup_L	−24 –48 72			76.09
Cerebellum_6_R	24 –48 -27	<.0001	631	120.91
Cerebellum_4_5_R	18 –51 -21			112.44
Precentral_R	39 –21 54	< .0001	1166	107.50
Parietal_Sup_R	21 –54 69			21.61
Supp_Motor_Area_R	9 –6 51			19.44
Frontal_Sup_L	−15 60 9	<.0001	908	33.41
ACC L	−6 45 –3			28.24
SupraMarginal_R	66 –21 36	.042	151	30.37
Postcentral_R	66 –6 18			14.80

The comparison between high-rated over low-rated pictures presented activations in bilateral precentral and postcentral gyri, bilateral superior parietal lobule, right cerebellum, left superior frontal gyrus, right supplementary motor area, left anterior cingulate gyrus, right supramarginal gyrus (FWE corrected).

**Fig. 2:**
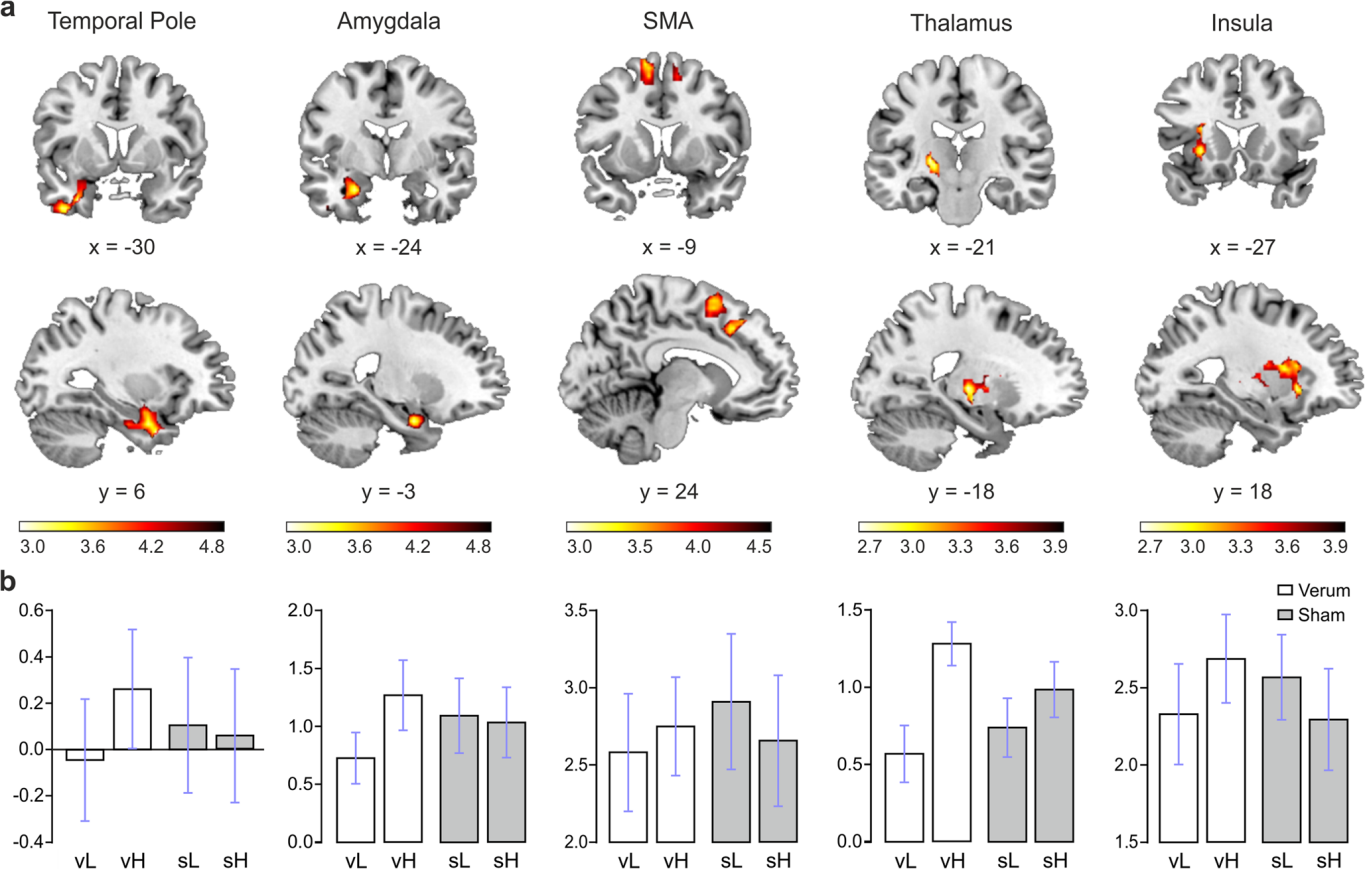
Whole Brain Analysis. **a**. Interaction between verum vs sham stimulation and high vs low ratings. **b**. Bar graphs indicate mean beta values with standard error of the mean (SEM; white for verum stimulation, grey for sham stimulation; SMA = supplementary motor area; vH = verum stimulation, high ratings; vL = verum stimulation, low ratings; sH = sham stimulation, high ratings; sL: sham stimulation, low ratings)

A significant interaction between the two factors was found in the left middle temporal pole, left amygdala, left inferior temporal gyrus, bilateral supplementary motor areas, left medial frontal gyrus, left thalamus and left insula (FWE corrected; Fig.2; Table 4).

**Table 4:** Enhanced fMRI signals for the interaction between session (verum and sham) and ratings (high and low)

Anatomical area	cluster level			voxel level	
***Whole brain analysis***	Coordinates	FWEc	Cluster size	FWEc	Cluster size
Temporal Pole Mid L	–30 6 –33	.020	225		4.92
Amygdala_L	−24 –3 –24				4.86
Temporal Inf L	−39 3 –39				4.43
Supp Motor Area R	6 21 54	.001	367		4.24
Frontal Sup Medial L	−9 24 42				4.19
Supp Motor Area L	−12 9 66				3.87
Thalamus L	−21 –18 3	.013	248		3.90
Insula L	−27 18 –3				3.49
***ROI analysis***					
Midbrain R	9 –18 –9		28	.014	4.19
Hippocampus L	−24 –6 –24		16	.014	4.34
Frontal_med_orb L	0 36 –15		14	.009	3.99

#### ROI analysis

**Fig. 3:**
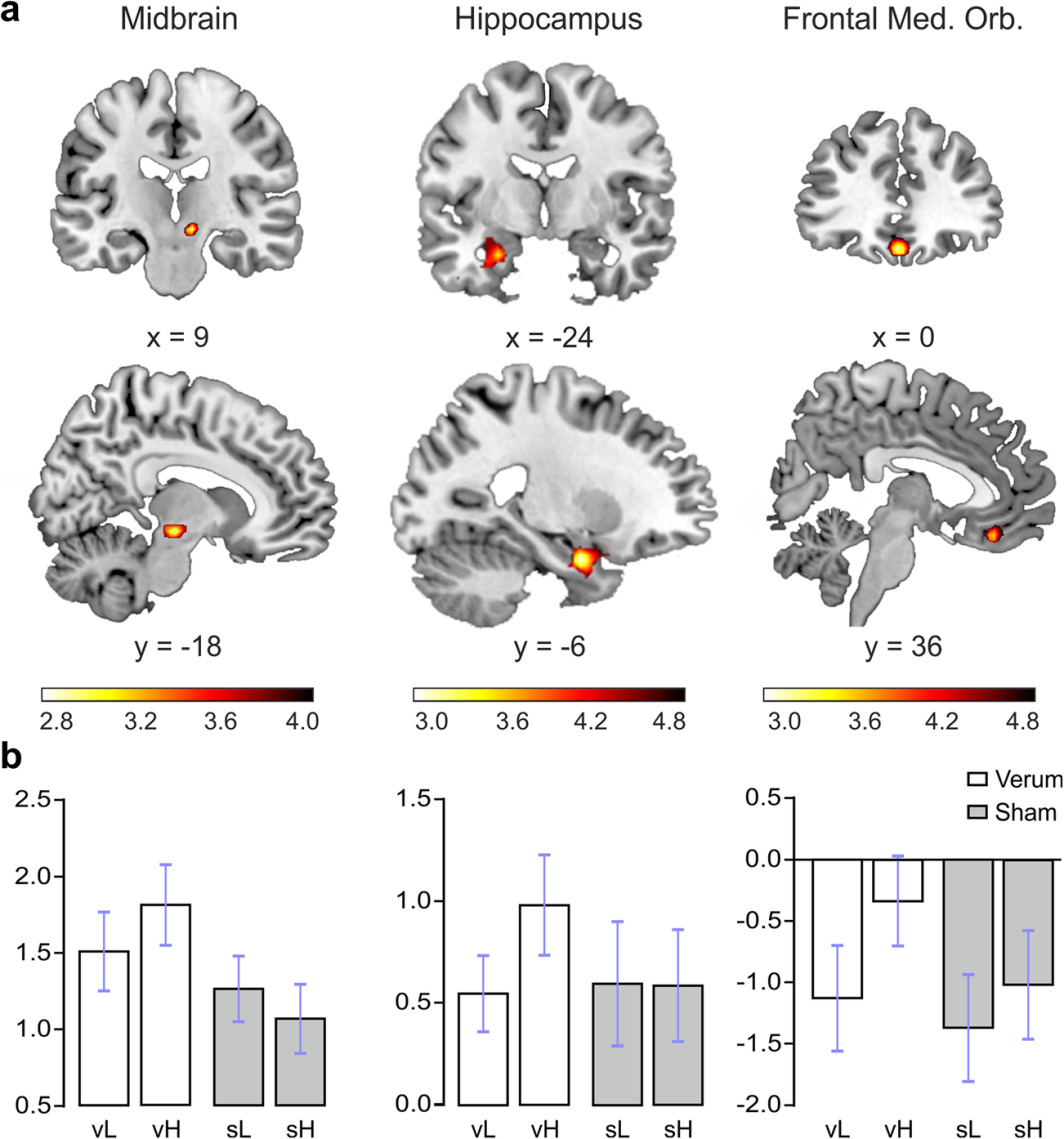
ROI analysis. Stimulation x rating interactions. A. Illustration of the significant ROIs B. Mean beta values with SEM. Please notice the differences in scale for the three ROIs. (vH = verum stimulation, high ratings; vL = verum stimulation, low ratings; sH = sham stimulation, high ratings; sL: sham stimulation, Low ratings)

In addition, the ROI analysis revealed significant stimulation x rating interactions in the right midbrain, left middle frontal gyrus (orbital), and left hippocampus (FWE corrected at the voxel level; Fig.3; Table 4). There were no significant interaction effects in the hypothalamus and nucleus accumbens.

## Discussion

The main goal of this study was to assess the potential effect of tVNS on the neural processing of food-related stimuli using fMRI. To pinpoint the effects of tVNS on the valuation of food-related stimuli, we asked participants to rate the food items and then assessed the interaction effect of stimulation and rating on brain activations. We found interaction effects in a number of brain areas including the left middle temporal pole, left amygdala, left inferior temporal gyrus, bilateral supplementary motor areas, left medial frontal gyrus, left thalamus, left insula, right midbrain, left OFC and left hippocampus. This is clear evidence for a specific modulatory effect of tVNS on the processing of food items. These results reveal an effect of tVNS outlasting the actual stimulation period. Indeed, in the present study, participants received only 1 h of stimulation prior to entering the scanner but were not stimulated while in the scanner.

Greater activation in the aforementioned areas were found for the verum tVNS session and high-rated pictures. Compared to viewing pictures of nonfoods, activations of the fusiform gyrus, OFC and middle insula have been commonly reported in response to food pictures (van der Laan et al. 2011 for a review). Therefore, our pattern of activations is in accordance with the network subserving food processing, including temporal visual integration areas (such as the temporal pole) and the salience network.

Behaviorally, there was a tendency to rate the food pictures as less pleasant for verum compared to sham stimulation. This result is in agreement with the main hypothesis about the satiety-mimicking effects of VNS (and tVNS). In contrast, there were no differences between sessions concerning the amount of consumed food. The lack of differences may be due to the short period of stimulation. In fact, animal (Roslin and Kurian 2001; Val-Laillet et al. 2010; Gil et al. 2011) and human studies (Burneo et al. 2002; Pardo et al. 2007) reporting weight loss and a decrease of food consumption as a consequence of VNS have found these effects after chronic stimulation. On the other hand, Bodenlos et al. (2007a; 2007b)) found a decrease in sweet food cravings in acute VNS in depressed patients. Also, Bodenlos et al. (2014) found that lean individuals (but not obese individuals) consumed fewer calories when the device was on than when the device was off. However, the participants were patients with implanted VNS devices, which were kept on or turned off depending on the session. This might have different implications from the comparison between verum and sham stimulation in healthy participants without previous VNS. They concluded that obese patients may be more resistant to satiety signals through vagal afferents.

As previously argued, ingestive behavior is mediated by interacting homeostatic and non-homeostatic pathways (for reviews see Berthoud 2006; Lutter and Nestler 2009). Homeostatic hormonal signals control for energy balance, while external cues such as high palatable food may also drive food intake even in absence or biological need. There is interaction of these two systems as the perception of food reward and palatability is modulated by homeostatic signals. Accordingly, in hunger state foods are perceived as more rewarding or desirable while, after eating, meal-induced satiety signals are sent through vagal afferents and other pathways and there is a decrease in the hedonic properties of food (Gautier et al. 2000; Mehta et al. 2012; Morton et al. 2014). Studies assessing brain responses to food pictures depending on the hungry/satiated state show that OFC and amygdala are activated more strongly during the hungry state compared with the satiated state, and decreased activations in limbic and paralimbic areas are reported as a consequence of satiation (Tataranni et al. 2002; Gautier et al. 2000; Baicy et al. 2007; Mehta et al., 2012). On the other hand, Baicy et al. (2007) reported larger activations in the prefrontal cortex linked to satiety. In the prefrontal cortex, OFC has been reported as a key region in the coding of the reward value, the expected reward value and the subjective pleasantness of foods and other reinforcers (see Kringelbach 2005 for a review). Moreover, it has been suggested to play a crucial role as an integration site between hunger and satiety signals (Rolls 2007 for a review). Results from animal studies have shown that connections of the amygdala with orbitofrontal and anterior temporal associative visual areas are robust and bidirectional (Ghashghaei and Barbas 2002; Rolls and Grabenhorst 2008 for a review). In addition, this network also includes the thalamus, which receives projections from the amygdala and inferior temporal cortex and, in turn, projects to the OFC (Rolls and Grabenhorst 2008). The OFC also receives projections from the hypothalamus, ventral-tegmental area, nucleus coeruleus, the raphe nuclei (Morecraft et al. 1992). Importantly, our results show a differential activation for high-rated food pictures in the left amygdala, OFC and also in the temporal pole. This visual integration area has been identified as an important area in emotional saliency of visual stimuli (Blaizot et al. 2010) and found to present differential-related activations between states of hunger and satiety (Mehta et al. 2012). Both temporal pole and OFC respond to emotional visual stimuli as reported by Royet et al. (2000). Our results for the main effects of stimulation show increased activations in left inferior and superior parietal lobe. These areas have been previously related to food stimuli with high hedonic value together with the visual integration area in the temporal pole and hippocampus (Baicy et al. 2007; Tomasi et al. 2014; Cornier et al. 2007).

In addition to the discussed regions, we also found differential activations in the left insula, hippocampus and bilateral SMA for the interaction between stimulation and ratings to the food pictures. Evidence from animal studies of the gustatory pathway (Rolls 2016 for a review) has shown that gustatory information reaches the NTS. The NTS projects to the taste thalamus, which then projects to the taste primary cortex in the anterior insula. From the insula, projections are sent to OFC and amygdala, which both sent projections to the hypothalamus and ventral striatum. In contrast to the areas coding the reward value of the food, the brain response to taste in the primary taste cortex seems to be little affected by satiety (Rolls 2016). This network for taste processing in animals coincides with the network subserving food pictures processing as described in neuroimaging studies. Appetizing foods have been also found to recruit the insula (Simmons et al. 2005; Tomasi et al. 2014), a result that is in agreement with our findings. Interestingly, the dorsal mid-insula is believed to integrate gustatory and interoceptive information (Avery et al. 2017).

Current results are also in accordance with previous studies demonstrating a stimulation effect by using invasive VNS, yet without any specificity for food processing. For instance, Bohning et al. (2001) performed an fMRI study with patients with epilepsy receiving VNS with implanted devices. In line with our results, they reported BOLD fMRI activations in OFC, left temporal cortex and left amygdala among other areas including parieto-occipital cortex and hypothalamus. Likewise, Lomarev et al. (2002) compared the effects of different frequencies of stimulation in an fMRI study with depressed patients receiving VNS. They found increased activations in the OFC and thalamus, the frontal pole, hypothalamus and left pallidum in the high stimulation frequency (20 Hz), which is the most analogous stimulation to the one given to our participants (25 Hz).

Finally, studies using tVNS have shown comparable stimulation-related activations to invasive VNS in the brain areas receiving vagal afferent projections. Kraus et al. (2007), in an fMRI study where participants received tVNS inside the scanner, found increased activations in the insula, thalamus and precentral gyrus. On the other hand, they reported deactivations in the amygdala, hippocampus, parahippocampal gyrus and the middle and superior temporal gyrus. In agreement with our results, Dietrich et al. (2008) reported activations in the brainstem (specifically in the locus coeruleus), left and right thalamus, left prefrontal cortex, left insula, right and left postcentral gyri and left posterior cingulated gyrus in the comparison between fMRI activations during tVNS stimulation in the left tragus and baseline. Frangos et al. (2015) found that cymba conchae stimulation in comparison with baseline and sham earlobe stimulation produced bilateral activations in the insula, paracentral lobule and anterior thalamic nuclei, and contralateral activity in the nucleus accumbens and amygdala. With a similar methodology, Yakunina et al. (2017) found that stimulation in the cymba conchae resulted in the strongest activation of the NTS and locus coeruleus as compared to the stimulation in the inner tragus and in the ear canal.

One limitation of this study is that 1 h of tVNS might not be sufficient in order to induce satiety sensation and, consequently, to be reflected in a significant decrease in food consumption. As previously addressed in the introduction section, the rationale behind the design for the current study was based on previous research showing VNS satiety-mimicking effects. The use of a non-invasive approach would be appealing and easy to implement in obese patients. Indeed, we have replicated previous results in patients with implanted VNS devices showing brain activations in vagal afferents (Bohning et al. (2001; Lomarev et al. 2002), and also in healthy volunteers receiving tVNS inside the scanner (Kraus et al., 2007). However, due to the sort stimulation period and the interaction effects found between verum and sham stimulation and high and low liking ratings, the satiation effect is presumed, and we could not discard the influence of other factors such as motivation or food preference. We hypothesize that an increase of stimulation time is needed to assess possible changes in short- and long-term control systems of food intake, and also crucial in order to find effects on weight loss.

As we did not differentiate our pictures into sweet and salty varieties, we could not replicate the previously reported decrease in craving for sweet foods. Likewise, we could not assess possible differential effects depending on caloric content and food palatability, which might have influenced current interaction results based on behavioral ratings. These issues might be addressed in future research.

Despite these limitations, the current findings showing differences in brain activations in key areas from the food homeostatic, gustatory and reward systems with only 1 h of tVNS are encouraging, and further investigation with healthy and obese patients receiving tVNS will help in identifying the best procedure for its potential therapeutic benefits.

## References

[CR1] Alves MB, Dalle Molle R, Desai M, Ross MG, Silveira PP (2015). Increased palatable food intake and response to food cues in intrauterine growth-restricted rats are related to tyrosine hydroxylase content in the orbitofrontal cortex and nucleus accumbens. Behavioural Brain Research.

[CR2] Avery JA, Gotts SJ, Kerr KL, Burrows K, Ingeholm JE, Bodurka J (2017). Convergent gustatory and viscerosensory processing in the human dorsal mid-insula. Human Brain Mapping.

[CR3] Baicy K, London ED, Monterosso J, Wong M-L, Delibasi T, Sharma A, Licinio J (2007). Leptin replacement alters brain response to food cues in genetically leptin-deficient adults. Proceedings of the National Academy of Sciences.

[CR4] Berthoud H-R (2006). Homeostatic and non-homeostatic pathways involved in the control of food intake and energy balance. Obesity.

[CR5] Berthoud HR (2008). The vagus nerve, food intake and obesity. Regulatory Peptides.

[CR6] Blaizot X, Mansilla F, Insausti AM, Constans JM, Salinas-Alamán A, Pró-Sistiaga P (2010). The human parahippocampal region: I. temporal pole cytoarchitectonic and MRI correlation. Cerebral Cortex.

[CR7] Bodenlos JS, Kose S, Borckardt JJ, Nahas Z, Shaw D, O’Neil PM, Pagoto SL (2007). Vagus nerve stimulation and emotional responses to food among depressed patients. Journal of Diabetes Science and Technology.

[CR8] Bodenlos JS, Kose S, Borckardt JJ, Nahas Z, Shaw D, O’Neil PM, George MS (2007). Vagus nerve stimulation acutely alters food craving in adults with depression. Appetite.

[CR9] Bodenlos JS, Schneider KL, Oleski J, Gordon K, Rothschild A, Pagoto SL (2014). Vagus nerve stimulation and food intake: Effect of body mass index. Journal of Diabetes Science and Technology.

[CR10] Bohning DE, Lomarev MP, Denslow S, Nahas Z, Shastri A, George MS (2001). Feasibility of vagus nerve stimulation-synchronized blood oxygenation level-dependent functional MRI. Investigative Radiology.

[CR11] Bradley R (2007). The role of the nucleus of the solitary tract in gustatory processing.

[CR12] Bray GA (2000). Afferent signals regulating food intake. Proceedings of the Nutrition Society.

[CR13] Burneo JG, Faught E, Knowlton R, Morawetz R, Kuzniecky R (2002). Weight loss associated with vagus nerve stimulation. Neurology.

[CR14] Castro DC, Cole SL, Berridge KC (2015). Lateral hypothalamus, nucleus accumbens, and ventral pallidum roles in eating and hunger: Interactions between homeostatic and reward circuitry. Frontiers in Systems Neuroscience.

[CR15] Cabanac M (1979). Sensory pleasure. The Quarterly Review of Biology.

[CR16] Chae JH, Nahas Z, Lomarev M, Denslow S, Lorberbaum JP, Bohning DE, George MS (2003). A review of functional neuroimaging studies of vagus nerve stimulation (VNS). Journal of Psychiatric Research.

[CR17] Cimpianu CL, Strube W, Falkai P, Palm U, Hasan A (2017). Vagus nerve stimulation in psychiatry: A systematic review of the available evidence. Journal of Neural Transmission.

[CR18] Clancy JA, Mary DA, Witte KK, Greenwood JP, Deuchars SA, Deuchars J (2014). Non-invasive Vagus nerve stimulation in healthy humans reduces sympathetic nerve activity. Brain Stimulation.

[CR19] Cornier M, Kaenel SS, Bessesen DH, Tregellas JR (2007). Effects of overfeeding on the neuronal response to visual food cues. The American Journal of Clinical Nutrition.

[CR20] de Lartigue G (2016). Role of the vagus nerve in the development and treatment of diet-induced obesity. Journal of Physiology.

[CR21] de Lartigue G, Diepenbroek C (2016). Novel developments in vagal afferent nutrient sensing and its role in energy homeostasis. Current Opinion in Pharmacology.

[CR22] Dietrich S, Smith J, Scherzinger C, Hofmann-Preiß K, Freitag T, Eisenkolb A, Ringler R (2008). A novel transcutaneous vagus nerve stimulation leads to brainstem and cerebral activations measured by functional MRI / Funktionelle Magnetresonanztomographie zeigt Aktivierungen des Hirnstamms und weiterer zerebraler Strukturen unter transkutaner Vagusne. Biomedizinische Technik/Biomedical Engineering.

[CR23] Ellrich J (2011). Transcutaneous vagus nerve stimulation. European Neurological Review.

[CR24] Ferrario CR, Labouèbe G, Liu S, Nieh EH, Routh VH, Xu S, O’Connor EC (2016). Homeostasis meets motivation in the Battle to control food intake. The Journal of Neuroscience.

[CR25] Fornai F, Ruffoli R, Giorgi FS, Paparelli A (2011). The role of locus coeruleus in the antiepileptic activity induced by vagus nerve stimulation. European Journal of Neuroscience.

[CR26] Frangos E, Ellrich J, Komisaruk BR (2015). Non-invasive access to the vagus nerve central projections via electrical stimulation of the external ear: FMRI evidence in humans. Brain Stimulation.

[CR27] Gautier JF, Chen K, Salbe AD, Bandy D, Pratley RE, Heiman M, Ravussin E, Reiman EM, Tataranni PA (2000). Differential brain responses to satiation in obese and lean men. Diabetes.

[CR28] George MS, Sackeim HA, Rush A, Marangell LB, Nahas Z, Husain MM (2000). Vagus nerve stimulation: A new tool for brain research and therapy∗. Biological Psychiatry.

[CR29] Ghashghaei HT, Barbas H (2002). Pathways for emotion: Interactions of prefrontal and anterior temporal pathways in the amygdala of the rhesus monkey. Neuroscience.

[CR30] Gil K, Bugajski A, Thor P (2011). Electrical vagus nerve stimulation decreases food consumption and weight gain in rats fed a high-fat diet. Journal of Physiology and Pharmacology.

[CR31] Groves DA, Bowman EM, Brown VJ (2005). Recordings from the rat locus coeruleus during acute vagal nerve stimulation in the anaesthetised rat. Neuroscience Letters.

[CR32] Groves DA, Brown VJ (2005). Vagal nerve stimulation: A review of its applications and potential mechanisms that mediate its clinical effects. Neuroscience and Biobehavioral Reviews.

[CR33] Henry TR (2002). Therapeutic mechanisms of vagus nerve stimulation. Neurology..

[CR34] Kringelbach ML (2005). The human orbitofrontal cortex: Linking reward to hedonic experience. Nature reviews. Neuroscience.

[CR35] Huang F, Dong J, Kong J, Wang H, Meng H, Spaeth RB, Camhi S, Liao X, Li X, Zhai X, Li S, Zhu B, Rong P (2014). Effect of transcutaneous auricular vagus nerve stimulation on impaired glucose tolerance: A pilot randomized study. BMC Complementary and Alternative Medicine.

[CR36] Jean A (1991). The nucleus tractus solitarius: Neuroanatomic, neurochemical and functional aspects. Archives of Biochemistry and Biophysics.

[CR37] Krahl SE, Clark KB (2012). Vagus nerve stimulation for epilepsy: A review of central mechanisms. Surgical Neurology International.

[CR38] Kraus T, Hösl K, Kiess O, Schanze A, Kornhuber J, Forster C (2007). BOLD fMRI deactivation of limbic and temporal brain structures and mood enhancing effect by transcutaneous vagus nerve stimulation. Journal of Neural Transmission.

[CR39] Li H, Zhang J-B, Xu C, Tang Q-Q, Shen W-X, Zhou J-Z, Chen JD, Wang YP (2015). Effects and mechanisms of auricular vagus nerve stimulation on high-fat-diet—Induced obese rats. Nutrition.

[CR40] Lomarev M, Denslow S, Nahas Z, Chae JH, George MS, Bohning DE (2002). Vagus nerve stimulation (VNS) synchronized BOLD fMRI suggests that VNS in depressed adults has frequency/dose dependent effects. Journal of Psychiatric Research.

[CR41] Lutter M, Nestler EJ (2009). Homeostatic and hedonic signals interact in the regulation of food intake. The Journal of Nutrition.

[CR42] Mehta S, Melhorn SJ, Smeraglio A, Tyagi V, Grabowski T, Schwartz MW, Schur EA (2012). Regional brain response to visual food cues is a marker of satiety that predicts food choice. American Journal of Clinical Nutrition.

[CR43] Morecraft RJ, Geula C, Mesulam M (1992). Cytoarchitecture and neural afferents of orbitofrontal cortex in the brain of the monkey. The Journal of Comparative Neurology.

[CR44] Morton GJ, Cummings DE, Baskin DG, Barsh GS, Schwartz MW (2006). Central nervous system control of food intake and body weight. Nature.

[CR45] Morton GJ, Meek TH, Schwartz MW (2014). Neurobiology of food intake in health and disease. Nature Reviews Neuroscience.

[CR46] Page AJ, Kentish SJ (2017). Plasticity of gastrointestinal vagal afferent satiety signals. Neurogastroenterology and Motility.

[CR47] Pardo JV, Sheikh SA, Kuskowski MA, Surerus-Johnson C, Hagen MC, Lee JT, Rittberg BR, Adson DE (2007). Weight loss during chronic, cervical vagus nerve stimulation in depressed patients with obesity: An observation. International Journal of Obesity.

[CR48] Peuker ET, Filler TJ (2002). The nerve supply of the human auricle. Clinical Anatomy.

[CR49] Rolls ET (2007). Understanding the mechanisms of food intake and obesity. Obesity Reviews.

[CR50] Rolls ET (2016). Functions of the anterior insula in taste, autonomic, and related functions. Brain and Cognition.

[CR51] Rolls ET, Grabenhorst F (2008). The orbitofrontal cortex and beyond: From affect to decision-making. Progress in Neurobiology.

[CR52] Roslin M, Kurian M (2001). The use of electrical stimulation of the vagus nerve to treat morbid obesity. Epilepsy and Behavior.

[CR53] Royet JP, Zald D, Versace R, Costes N, Lavenne F, Koenig O, Gervais R (2000). Emotional responses to pleasant and unpleasant olfactory, visual, and auditory stimuli: A positron emission tomography study. The Journal of neuroscience : the official journal of the Society for Neuroscience.

[CR54] Ruffoli R, Giorgi FS, Pizzanelli C, Murri L, Paparelli A, Fornai F (2011). The chemical neuroanatomy of vagus nerve stimulation. Journal of Chemical Neuroanatomy.

[CR55] Rush AJ, Gullion CM, Basco MR, Jarrett RB, Trivedi MH (1996). The inventory of depressive symptomatology (IDS): Psychometric properties. Psychological Medicine.

[CR56] Safi S, Ellrich J, Neuhuber W (2016). Myelinated axons in the auricular branch of the human Vagus nerve. Anatomical Record.

[CR57] Saper C, Loewy A (1980). Efferent connections of the parabrachial nucleus in the rat. Brain Research.

[CR58] Sawchenko P (1983). Central connections of the sensory and motor nuclei of the vagus nerve. Journal of the Autonomic Nervous System.

[CR59] Shechter A, Schwartz GJ (2018). Gut–brain nutrient sensing in food reward. Appetite.

[CR60] Simmons WK, Martin A, Barsalou LW (2005). Pictures of appetizing foods activate gustatory cortices for taste and reward. Cerebral Cortex.

[CR61] Stefan H, Kreiselmeyer G, Kerling F, Kurzbuch K, Rauch C, Heers M, Kasper BS, Hammen T, Rzonsa M, Pauli E, Ellrich J, Graf W, Hopfengärtner R (2012). Transcutaneous vagus nerve stimulation (t-VNS) in pharmacoresistant epilepsies: A proof of concept trial. Epilepsia.

[CR62] Stunkard AJ, Messick S (1985). The three-factor eating questionnaire to measure dietary restraint, disinhibition and hunger. Journal of Psychosomatic Research.

[CR63] Tataranni PA, Gautier J-F, Chen K, Uecker A, Bandy D, Salbe AD, Pratley RE, Lawson M, Reiman EM, Ravussin E (2002). Neuroanatomical correlates of hunger and satiation in humans using positron emission tomography. Proceedings of the National Academy of Sciences.

[CR64] Tomasi D, Wang G-J, Wang R, Caparelli EC, Logan J, Volkow ND (2014). Overlapping patterns of brain activation to food and cocaine cues in cocaine abusers. Human Brain Mapping.

[CR65] Val-Laillet D, Biraben A, Randuineau G, Malbert CH (2010). Chronic vagus nerve stimulation decreased weight gain, food consumption and sweet craving in adult obese minipigs. Appetite.

[CR66] Van Bockstaele EJ, Peoples J, Telegan P (1999). Efferent projections of the nucleus of the solitary tract to peri-locus coeruleus dendrites in rat brain: Evidence for a monosynaptic pathway. Journal of Comparative Neurology.

[CR67] van der Laan LN, de Ridder DTD, Viergever MA, Smeets PAM (2011). The first taste is always with the eyes: A meta-analysis on the neural correlates of processing visual food cues. NeuroImage.

[CR68] SPSS Inc. Released (2009). PASW statistics for windows, version 18.0.

[CR69] Strien TV, Frijters JE, Bergers GP, Defares PB (1986). The Dutch eating behavior questionnaire (DEBQ) for assessment of restrained, emotional, and external eating behavior. International Journal of Eating Disorders.

[CR70] Tzourio-Mazoyer, N., Landeau, B., Papathanassiou, D., Crivello, F., Etard, O., Delcroix, N., Mazoyer, B., & JoliotM. (2002). Automated anatomical labeling of activations in SPM using a macroscopic anatomical parcellation of the MNI MRI single-subject brain. *Neuroimage, 15*(1), 273–89. 10.1006/nimg.2001.0978. 10.1006/nimg.2001.0978 11771995

[CR71] Veldhuizen MG, Albrecht J, Zelano C, Boesveldt S, Breslin P, Lundström JN (2011). Identification of human gustatory cortex by activation likelihood estimation. Human Brain Mapping.

[CR72] Vonck K, Raedt R, Naulaerts J, De Vogelaere F, Thiery E, Van Roost D, Boon P (2014). Vagus nerve stimulation. . .25 years later! What do we know about the effects on cognition?. Neuroscience and Biobehavioral Reviews.

[CR73] Wang G-J, Yang J, Volkow ND, Telang F, Ma Y, Zhu W, Wong CT, Tomasi D, Thanos PK, Fowler S (2006). Gastric stimulation in obese subjects activates the hippocampus and other regions involved in brain reward circuitry. Proceedings of the National Academy of Sciences.

[CR74] Williams EK, Chang RB, Strochlic DE, Umans BD, Lowell BB, Liberles SD (2016). Sensory neurons that detect stretch and nutrients in the digestive system. Cell.

[CR75] Woods SC (1998). Signals that regulate food intake and energy homeostasis. Science.

[CR76] Yakunina N, Kim SS, Nam EC (2017). Optimization of transcutaneous Vagus nerve stimulation using functional MRI. Neuromodulation.

